# Differential cardiomyocyte transcriptomic remodeling during in vitro* Trypanosoma cruzi* infection using laboratory strains provides implications on pathogenic host responses

**DOI:** 10.1186/s41182-023-00552-6

**Published:** 2023-12-07

**Authors:** Katherine-Sofia Candray-Medina, Yu Nakagama, Masamichi Ito, Shun Nakagama, Evariste Tshibangu-Kabamba, Norihiko Takeda, Yuki Sugiura, Yuko Nitahara, Yu Michimuko-Nagahara, Natsuko Kaku, Yoko Onizuka, Carmen-Elena Arias, Maricela Mejia, Karla Alas, Susana Peña, Yasuhiro Maejima, Issei Komuro, Junko Nakajima-Shimada, Yasutoshi Kido

**Affiliations:** 1 Department of Parasitology, Graduate School of Medicine, Osaka Metropolitan University, 1-4-3 Asahi-machi, Abeno-ku, Osaka, 545-8585 Japan; 2 Research Center for Infectious Disease Sciences, Graduate School of Medicine, Osaka Metropolitan University, 1-4-3 Asahi-machi, Abeno-ku, Osaka, 545-8585 Japan; 3 Centro Nacional de Investigaciones Científicas de El Salvador (CICES), Ciudad Universitaria “Dr. Fabio Castillo Figueroa”, final avenida “Mártires del 30 de julio”, San Salvador, El Salvador; 4 Department of Cardiovascular Medicine, Graduate School of Medicine, The University of Tokyo, 7-3-1 Hongo, Bunkyo-ku, Tokyo, 113-0033 Japan; 5 Department of Advanced Clinical Science and Therapeutics, Graduate School of Medicine, The University of Tokyo, 7-3-1 Hongo, Bunkyo-ku, Tokyo, 113-0033 Japan; 6 Department of Cardiovascular Medicine, Graduate School of Medical and Dental Sciences, Tokyo Medical and Dental University, 1-5-45 Yushima, Bunkyo-ku, Tokyo, 113-8519 Japan; 7 Department of Internal Medicine, University of Mbujimayi, #1 avenue de l’Université, Dibindi, 225, Mbujimayi, Democratic Republic of Congo; 8 Division of Cardiology and Metabolism, Center for Molecular Medicine, Jichi Medical University, 3311-1 Yakushiji, Shimotsuke, 329-0498 Japan; 9 Center for Cancer Immunotherapy and Immunobiology, Kyoto University Graduate School of Medicine, Yoshida Nihonmatsucho, Sakyo-ku, Kyoto, 606-8501 Japan; 10 Department of Molecular and Cellular Parasitology, Graduate School of Health Sciences, Gunma University, 3-39-22 Showa-machi, Maebashi, Gunma 371-8514 Japan; 11 Departamento de Investigación, Hospital Nacional Especializado “Rosales”, Ministerio de Salud de El Salvador, Calle Arce No. 827, San Salvador, El Salvador; 12 International University of Health and Welfare, Minato-Ku, Tokyo, 107-8402 Japan; 13 Department of Frontier Cardiovascular Medicine, Graduate School of Medicine, The University of Tokyo, Bunkyo-ku, Tokyo, 113-8655 Japan

**Keywords:** *Trypanosoma cruzi*, Chagas disease, Transcriptome, HL-1, In vitro modeling, Dilated cardiomyopathy, Glutathione metabolism, Oxidative stress, Neglected tropical diseases

## Abstract

**Background:**

Chagas disease can lead to life-threatening cardiac manifestations. Regional factors, including genetic characteristics of circulating *Trypanosoma cruzi (T. cruzi)*, have attracted attention as likely determinants of Chagas disease phenotypic expression and Chagas cardiomyopathy (CCM) progression. Our objective was to elucidate the differential transcriptomic signatures of cardiomyocytes resulting from infection with genetically discrete *T. cruzi* strains and explore their relationships with CCM pathogenesis and progression.

**Methods:**

HL-1 rodent cardiomyocytes were infected with *T. cruzi* trypomastigotes of the Colombian, Y, or Tulahuen strain. RNA was serially isolated post-infection for microarray analysis. Enrichment analyses of differentially expressed genes (fold-change ≥ 2 or ≤ 0.5) highlighted over-represented biological pathways. Intracellular levels of reactive oxygen species (ROS) were compared between *T. cruzi*-infected and non-infected HL-1 cardiomyocytes.

**Results:**

We found that oxidative stress-related gene ontology terms (GO terms), ‘Hypertrophy model’, ‘Apoptosis’, and ‘MAPK signaling’ pathways (all with *P* < 0.01) were upregulated. ‘Glutathione and one-carbon metabolism’ pathway, and ‘Cellular nitrogen compound metabolic process’ GO term (all with *P* < 0.001) were upregulated exclusively in the cardiomyocytes infected with the Colombian/Y strains. Mean intracellular levels of ROS were significantly higher in the *T. cruzi*-infected cardiomyocytes compared to the non-infected (*P* < 0.0001).

**Conclusions:**

The upregulation of oxidative stress-related and hypertrophic pathways constitutes the universal hallmarks of the cardiomyocyte response elicited by *T. cruzi* infection. Nitrogen metabolism upregulation and glutathione metabolism imbalance may implicate a relationship between nitrosative stress and poor oxygen radicals scavenging in the unique pathophysiology of Chagas cardiomyopathy.

**Supplementary Information:**

The online version contains supplementary material available at 10.1186/s41182-023-00552-6.

## Introduction

Chagas disease, caused by *Trypanosoma cruzi* (*T. cruzi)* protozoan infection, affects more than 6 million people worldwide, with more than 70 million people at risk of infection [1–3]. In the absence of curative treatment, approximately 30% of those chronically infected will present Chagas cardiomyopathy (CCM) as a phenotypic outcome. CCM is a life-threatening condition characterized by myocardial dilatation, conduction abnormalities, and heart failure [4]. The molecular pathophysiology and indicators of CCM progression have been least described among other common forms of dilated cardiomyopathy, such as those related to ischemic/hypoxic stress (mitochondrial dysfunction and impaired calcium sensitivity of cardiomyocytes) or viral infections (viral RNA persistence and subsequent apoptotic loss of cardiomyocyte) [5–15].

The diverse genetic make-up of parasite virulence factors shall evoke unique host responses of variable magnitude [16–21], collectively affecting the phenotypic expression of CCM. Thus, the host response to specific *T. cruzi* genotypes may provide clues to the molecular pathophysiology of CCM progression [22–24]. The objective of the current study was to elucidate the transcriptomic signatures of the cardiomyocyte response to infection with genetically discrete *T. cruzi* strains namely, Colombian, Y, and Tulahuen to explore differential host responses and their relationships with CCM pathogenesis and progression.

## Materials and methods

### Cell lines and culture conditions

Mouse CH3/10T1/2, clone 8 murine fibroblasts (RRID: CVCL_0190, JCRB0003), obtained from the Japanese Collection of Research Bioresources Cell Bank (JCRB), were cultured in high-glucose Dulbecco’s modified DMEM medium (Fujifilm, Japan) supplemented with 10% fetal bovine serum and 1% penicillin/streptomycin (Fujifilm, Osaka, Japan). The cells were seeded in T-25 vent-cap flasks and incubated at 37 °C, under 5% CO_2_.

Mouse HL-1 cardiomyocytes (RRID: CVCL_0303) were cultured in Claycomb medium (Merck, Darmstadt, Germany) supplemented with 10% fetal bovine serum (Gibco, Maryland, USA), 0.1 mM norepinephrine (Sigma), 0.2 mM L-glutamine (Fujifilm, Osaka, Japan), and 1% penicillin/streptomycin (Fujifilm, Osaka, Japan). Cells were seeded on T-25 vent-cap flasks pre-coated with fibronectin (Sigma, St. Louis, MO, USA) and incubated at 37 °C, under 5% CO_2_.

*T. cruzi* epimastigotes of the Colombian, Y, and Tulahuen strains were obtained from NEKKEN Bio-Resource Center (NBRC) and cultured in LIT medium at 28 °C, under 5% CO_2_. Per strain, 1 × 10^8^ epimastigotes harvested in the exponential growth phase were subjected to metacyclogenesis, following a pre-established protocol [25]. Debris sedimentation was performed by centrifugation at 700 ×*g* for 5 min followed by trypomastigote collection of the supernatant at 1700 ×*g* for 10 min. Trypomastigotes were used to infect CH3/10T1/2 cells at a multiplicity of infection (MOI) 1:10. After 5–7 days, successful infection was confirmed by the presence of intracellular amastigotes on light microscopy. Cultured cell-derived trypomastigotes (CCdT), obtained from the supernatant of these stable cultures under the aforementioned centrifugation condition, were used for cardiomyocyte infection experiments.

### In vitro cardiomyocyte infection

The 6-well plates were seeded with HL-1 cardiomyocytes as described previously and incubated for 24 h to ensure full attachment and confluency. CCdT of each strain was collected to infect HL-1 cardiomyocytes at an MOI of 10:1. Mouse TNF-α (100 ng/mL; Sigma-Aldrich H8916, Missouri, USA) was added to the culture medium of the positive control wells [19, 21, 26–31]. CCdT were allowed to interact with the HL-1 cells for 24 or 48 h depending on the intended condition and were not washed during this interaction period. Non-infected conditions for both 24 and 48 h time points were prepared separately to control for unexpected changes during time course. Each condition was prepared in duplicates.

### Assessment of trypanosome infectivity by light microscopy

At 24- and 48-h post-infection (hpi), cells were fixed and stained with Diff-Quick solution (Sysmex, Kobe, Japan). For each cardiomyocyte infection condition, 'percentage of infected cells', as well as the 'intracellular amastigote count per infected cell', were determined by light microscopy to serve as surrogates of the strains infectivity rate. Eight microscopic fields were counted after staining. The results are representative of two separately performed experiments. Pairwise comparisons were performed with the Mann–Whitney test.

### Microarray analysis of differentially expressed genes (DEGs)

At 24 and 48 hpi, the culture medium was removed, and cells were gently washed with sterile PBS. Total RNA was isolated using the NucleoSpin RNA kit (Macherey–Nagel, Düren, Germany), according to the manufacturer’s instructions. RNA concentration and purity were assessed using Nanodrop 2000 (ThermoFisher, Wilmington, USA). RNA samples were analyzed using the Clariom S Assay for Mouse (Applied BioSystems, CA, USA) microarray platform to identify DEGs.

Data analysis was done on the Microarray Data Analysis Tool software version 3.2.0.0 (Filgen, Nagoya, Japan). Background signal was defined as any signal intensity values below the median of the signal intensity of a set of negative control probes on the microarray. The threshold for background level was determined as 43 for the infected conditions and 48 for the negative control at 24 hpi; 52 for the infected conditions and 48 for the negative control at 48 hpi. Valid gene probes were the set of probes which surpassed these thresholds of intensity in every infection condition at each time point. Probes not fulfilling the criteria were considered invalid and excluded. Therefore, 11,041 and 11,000 valid gene probes for each respective time point were included for further analyses. Fold change (FC) was defined as the ratio between gene expression level of the infected conditions (or the positive control) and the negative control. *P*-value (*P*) was calculated by the ‘Student’s t-test’ to determine the significance of FC of a gene per comparison. Multiple comparison-adjusted *P*-values were estimated by the Benjamini–Hochberg method using GraphPad Prism version 9.5.1 for Windows (GraphPad Software, Massachusetts, USA). FC ≥ 2 or ≤ 0.5, and *P* < 0.05 were used as the threshold to define DEGs.

FC of the valid genes shared across all cardiomyocyte infection conditions (total 10,784 genes) were used as input for the principal component analysis (PCA). Analysis and plotting were performed with R 4.1.0 software, and the ‘FactoMineR*’* and ‘Factoextra’ R packages [32–34]. The principal components 1 and 2, which together described 58.9% of the total variance in the dataset, were selected to plot the PCA results.

### Pathway and gene ontology enrichment analysis

Pathway and gene ontology (GO) category analysis was performed using the Microarray Data Analysis Tool software version 3.2.0.0 (Filgen, Nagoya, Japan). Pathways were enriched using the WikiPathways system [35].

Enrichment of both pathways and GO categories was performed using the gene sets (common to all three strains, or selectively shared between Colombian and Y strains) of upregulated and downregulated DEGs as input. The *P*-value for each pathway and GO category was calculated using the two-tailed Fisher’s exact test. Significantly (Z-score (*Z*) > 0, *P* < 0.01) enriched pathways and the top GO terms (up to ten, in the order of lowest *P*) were selected for discussion. Bar plots depicting GO and pathway enrichment were generated using the ‘GOplot’ R package [36].

### Assessment of reactive oxygen species (ROS) intracellular content

The 96-well plates were seeded to a 50% confluency with HL-1 cardiomyocytes as described previously and incubated for 24 h to ensure full attachment. CCdT of *T. cruzi* Y strain were used to infect HL-1 cardiomyocytes at an MOI of 10:1. Each condition was performed in triplicates. After 24 h, sterile PBS was used to wash non-internalized parasites. Cells were incubated with CellRox green (ThermoFisher, Wilmington, USA) at 10 µM for 30 min, then, gently washed and counterstained with Hoechst dye solution (Abcam, Cambridge, UK) at 4 µM for 15 min. Image acquisition was performed with a BZ-X700 series Keyence microscope (Keyence, Osaka, Japan). Image analyses were performed with the ImageJ software (National Institute of Health, USA) [37]. For each biological replicate, four cell images were randomly chosen to measure their fluorescence intensity for the CellRox green probe, and three random background measurements were also performed. Background fluorescence was subtracted from the cell measurements to calculate the cell fluorescence. Mann–Whitney test was used for group comparisons.

## Results

### Assessment of infectivity by strain

**Fig. 1:**
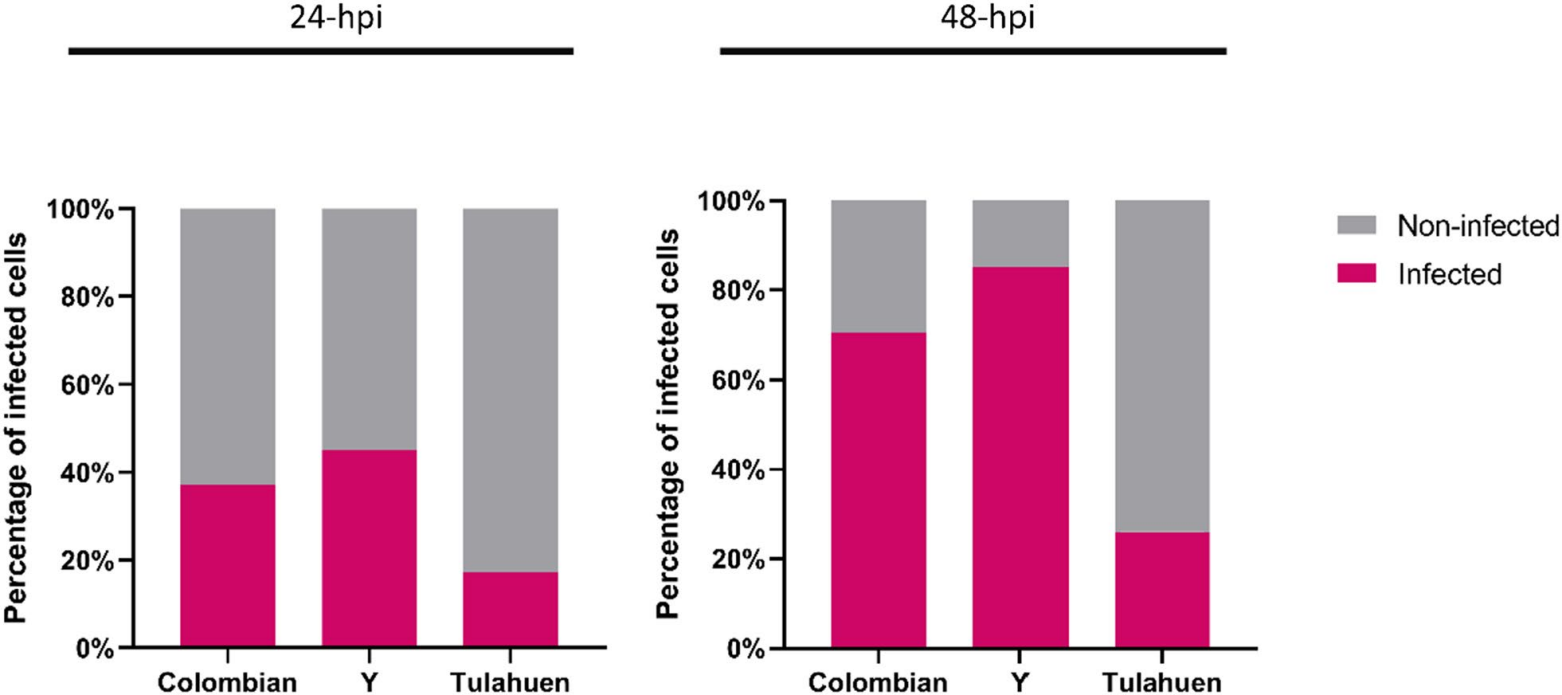
Difference in cardiomyocyte infectivity among *Trypanosoma cruzi* infection conditions. Percentage of *T*. *cruzi*-infected cells (containing intracellular amastigotes) at 24- and 48-h post-infection. *hpi* hours post-infection

**Fig. 2:**
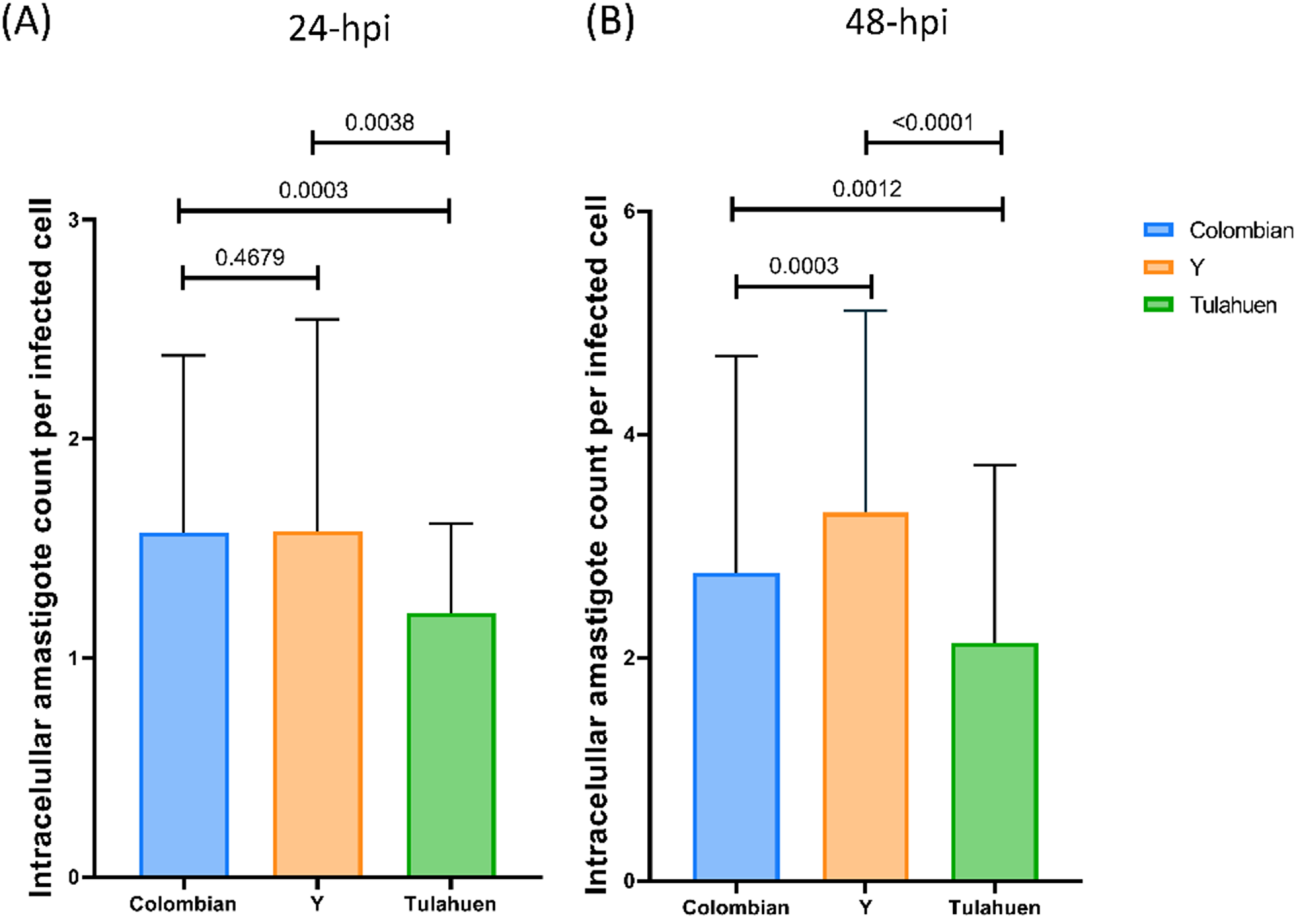
Cardiomyocyte infectivity among different *Trypanosoma cruzi* infection conditions. Intracellular amastigote counts per infected cell are plotted at **A** 24- and **B** 48-h post-infection. Pairwise comparisons were performed with Mann–Whitney test. Black horizontal bars indicate the mean and SD

At 24 hpi, Colombian, Y and Tulahuen infections showed 37.2%, 45.1%, and 17.2% infected cardiomyocytes, respectively (Fig. 1, left). A similar trend persisted at 48 hpi when the infected cardiomyocytes counted 70.5%, 85.2%, and 26.0% for Colombian, Y and Tulahuen infections, respectively (Fig. 1, right). At 24 hpi, each infected cell contained a mean (standard deviation): 1.6 (0.8), 1.6 (0.9), and 1.2 (0.4) intracellular amastigotes for Colombian-, Y-, and Tulahuen-infected conditions, respectively. A similar trend in differential infectivity persisted at 48 hpi, showing intracellular amastigote counts of 2.8 (1.9), 3.3 (1.8), and 2.1 (1.6) per Colombian, Y, and Tulahuen strain-infected cells, respectively. Pairwise comparisons found significant differences between Colombian and Y against Tulahuen strain (*P* < 0.01), in both 24 and 48 hpi. Distribution and exact *P* values are depicted in Fig. 2. A representative image from each infection condition is depicted in Additional file 2: Fig. S1.

### Host transcriptomic remodeling per infecting *T. cruzi* strain

**Fig. 3:**
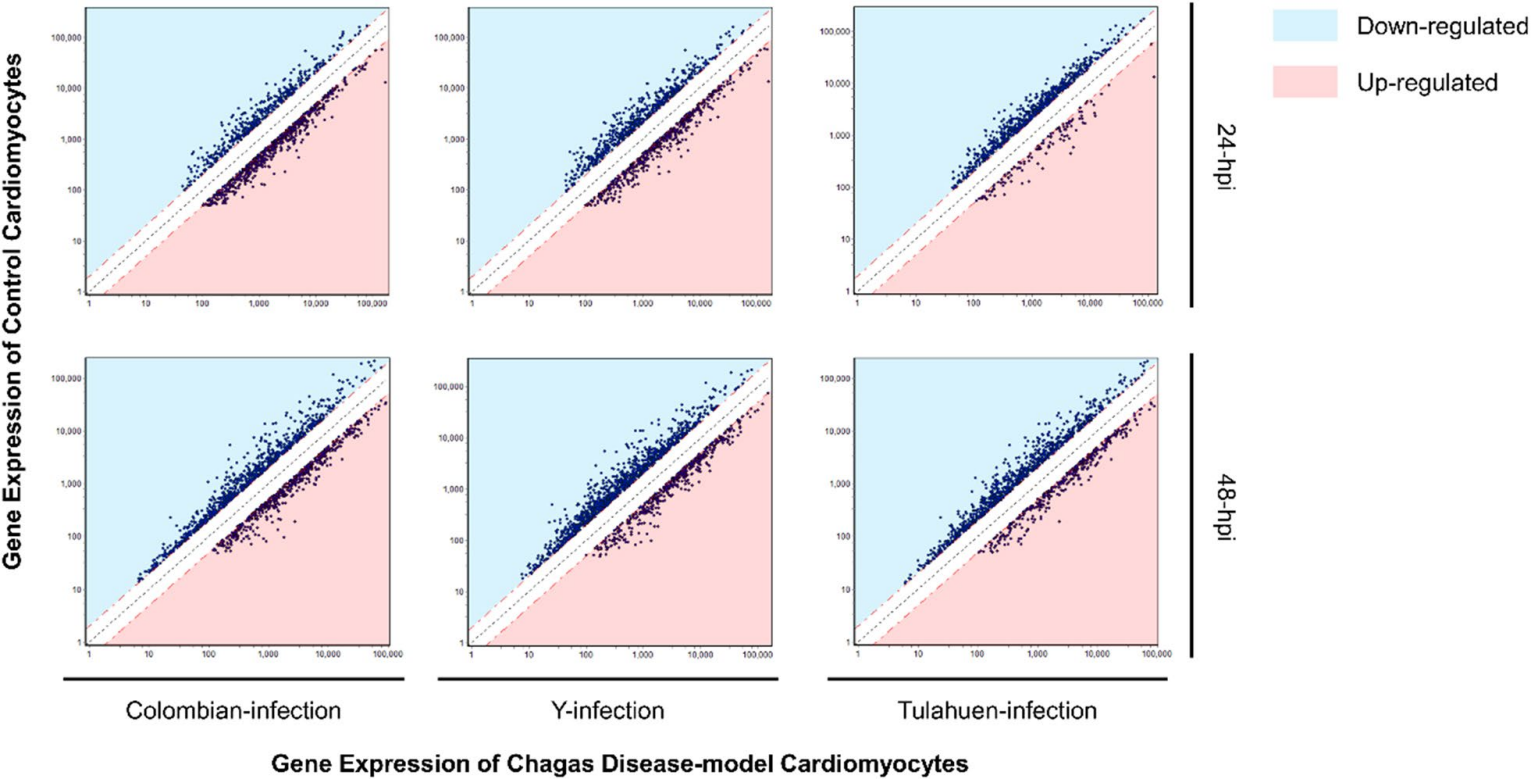
Remodeling of gene expression profiles in cardiomyocytes infected with three different *Trypanosoma cruzi* strains at two time points post-infection. Y axes indicate the gene expression level of the non-infected control cardiomyocyte, while X-axes indicate gene expression levels of *Trypanosoma cruzi*-infected cardiomyocytes. The black dashed line, and upper and lower red dashed lines show the line of identity, and the 0.5- and twofold change thresholds, respectively. Each plot represents a differentially expressed gene (DEG). Blue and pink shaded areas represent down- and up-regulated DEGs, respectively. *hpi* hours post-infection

**Fig. 4:**
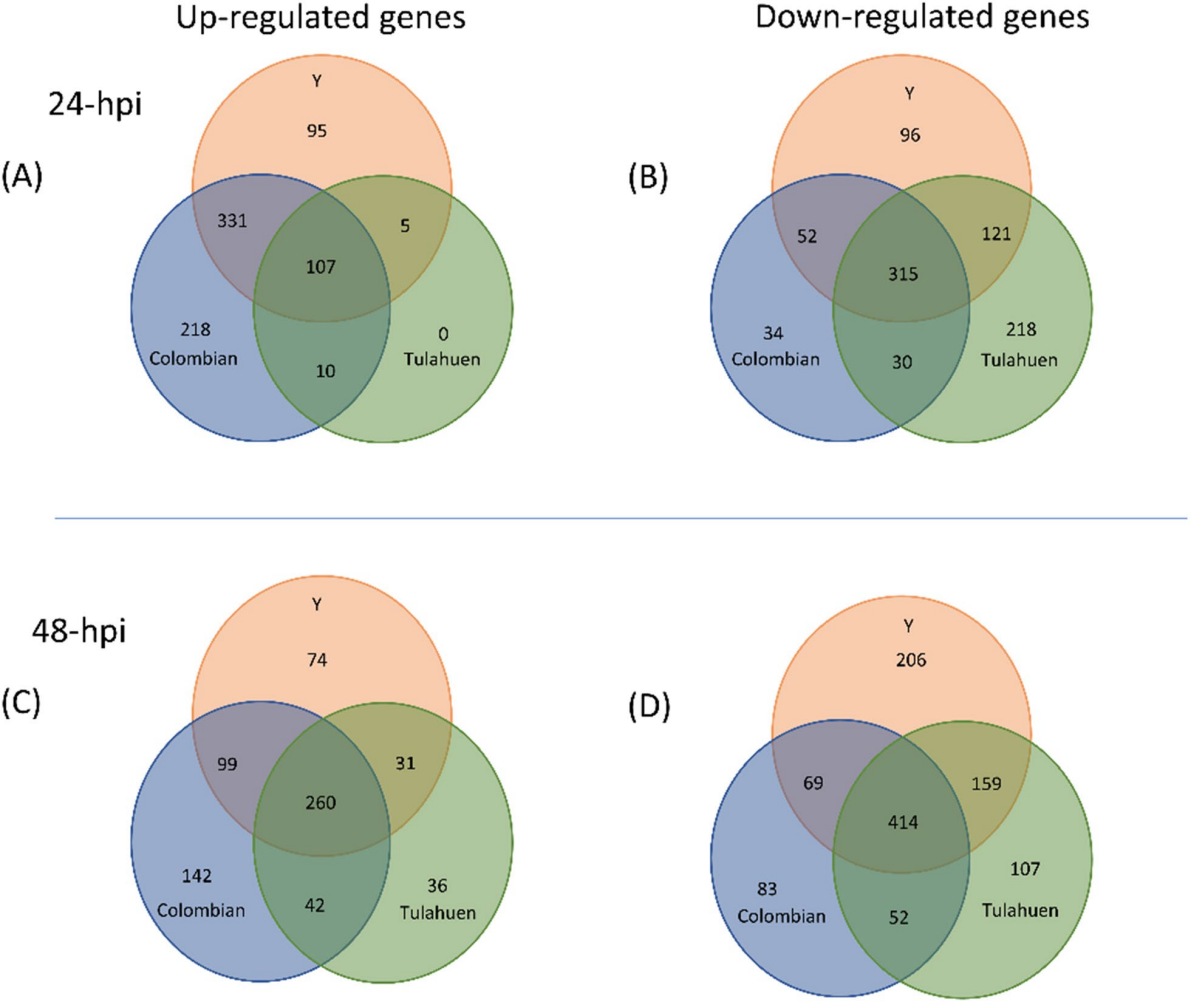
Identification of differentially expressed genes (DEGs) of the host cardiomyocyte upon *Trypanosoma cruzi* infection. Numbers of up- (left column) and down-regulated (right column) DEGs at 24- (upper row) and 48-h post-infection (lower row) are shown in the respective Venn diagrams. *hpi* hours post-infection

**Fig. 5:**
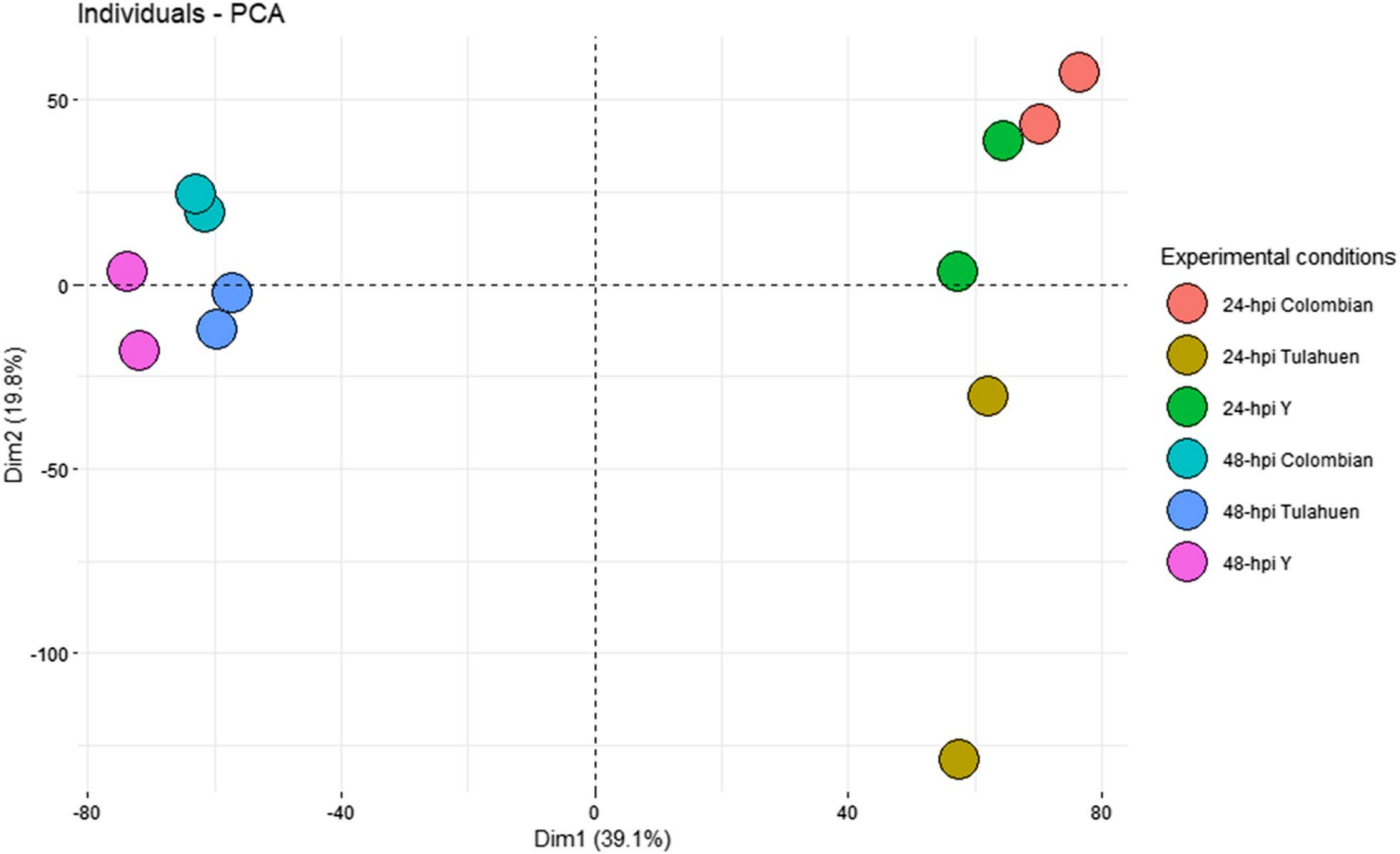
Principal component analysis plot of the transcriptional profile of the in vitro cardiomyocyte infection models. Principal component analysis plot, based on the fold-change (vs control) values of the 10,784 valid genes. Each dot represents a biological condition at 24- or 48-h post-infection. The principal components 1 and 2 together describe 58.9% of the total variance in the dataset. *Dim* dimension

In the Colombian-, Y-, and Tulahuen 24-h-infected conditions, 1097 (4.9%), 1122 (5.1%), and 806 (3.6%) DEGs were identified. 32 DEGs were identified in the TNF-α-treated control condition (Additional file 2: Fig. S2). The proportions of up- and down-regulated DEGs per cardiomyocyte infection condition are depicted in Fig. 3. Colombian and Y infections led to increased numbers of upregulated DEGs at 24 hpi. A similar trend persisted at 48 hpi but to a lesser extent. At 24 hpi, a significant overlap of upregulated DEGs (438 of 666 (66%) in Colombian-, or of 538 (81%) in Y-infection) was observed between Colombian and Y infection, of which 107 (24%) were also shared with Tulahuen-infection (Fig. 4A). Uniquely in Tulahuen-infection, downregulated genes over-represented the total DEGs at 24 hpi. A large proportion of downregulated DEGs (315 of 431 (73%) in Colombian-, 584 (54%) in Y-, and 684 (46%) in Tulahuen-infection) were shared among all infection conditions, suggesting a common pathway of cardiomyocyte functional deterioration (Fig. 4B). As apparent in the PCA plot, the Colombian- and Y-infection conditions were more closely related in terms of the gene expression pattern (Fig. 5), potentially reflecting the significant overlap of upregulated DEGs between the strains and the distinctive over-representation of downregulated DEG in Tulahuen-infection. At 24 hpi, the three infection conditions showed more discrete host cardiomyocyte gene expression patterns compared at 48 hpi. This indicated that 24 hpi would serve as a more favorable time frame to investigate the uniqueness of the host transcriptional remodeling triggered by each infecting *T. cruzi* strain. Not only were we more interested in the earlier determinants of CCM progression, but the PCA findings also supported our selection of the 24-hpi stage of the in vitro cardiomyocyte infection model as the main focus of the following analyses.

### The common cardiomyocyte response to *T. cruzi* infection

**Fig. 6:**
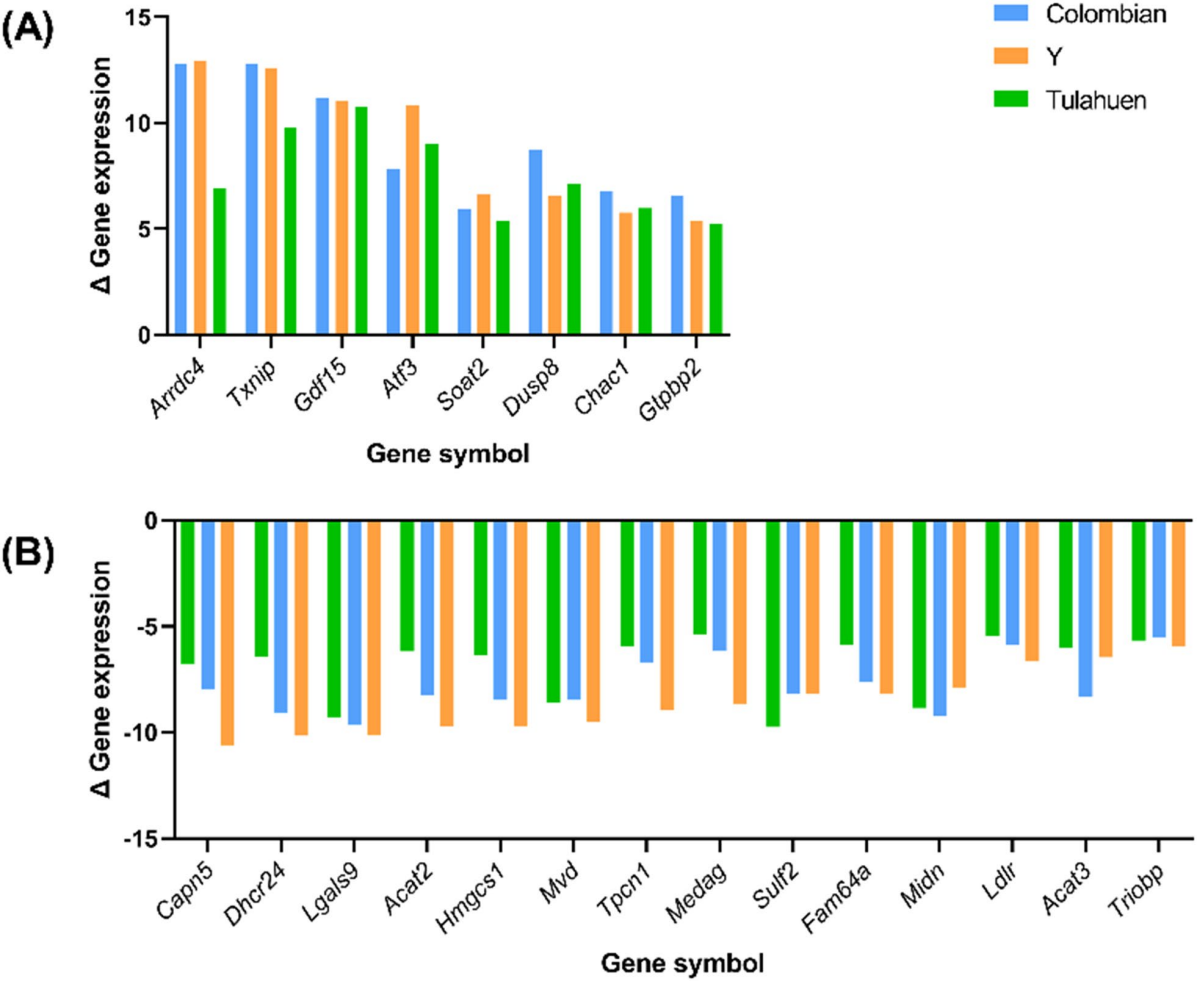
Top differentially expressed genes of the cardiomyocyte upon *Trypanosoma cruzi* infection. Extensively **A** upregulated (fold-change > 5) and **B** downregulated (fold-change < 0.2) genes of the cardiomyocyte at 24-h post-infection are listed. Δ Gene expression, the fold-change or ratio between gene expression level of the infected cardiomyocytes and the non-infected negative control. *Arrdc4* arrestin domain containing 4, *Txnip* thioredoxin interacting protein, *Gdf15* growth differentiation factor 15, *Atf3* activating transcription factor 3, *Soat2* sterol o-acyltransferase 2, *Dusp8* dual specificity phosphatase 8, *Chac1* cation transport regulator 1, *Gtpbp2* GTP binding protein 2, *Capn5* Calpain 5, *Dhcr24* 24-dehydrocholesterol reductase, *Lgals9* lectin galactose binding soluble 9, *Acat2* acetyl-coenzyme A acetyltransferase 2, *Hmgcs1* 3-hydroxy-3-methylglutaryl-Coenzyme A synthase 1, *Mvd* mevalonate (diphospho) decarboxylase, *Tpcn1* two pore channel 1, *Medag* mesenteric estrogen dependent adipogenesis, *Sulf2* sulfatase 2, *Fam64a* family with sequence similarity 64 member A, *Midn* midnolin, *Ldlr* low density lipoprotein receptor, *Acat3* acetyl-coenzyme A acetyltransferase 3, *Triobp* TRIO and F-actin binding protein

Of the 107 commonly upregulated genes, eight had FC ≥ 5. The top upregulated genes were Arresting Domain Containing 4 (*Arrdc4*), thioredoxin interacting protein (*Txnip*), and growth differentiation factor 15 (*Gdf15*). (Fig. 6 A).

**Fig. 7:**
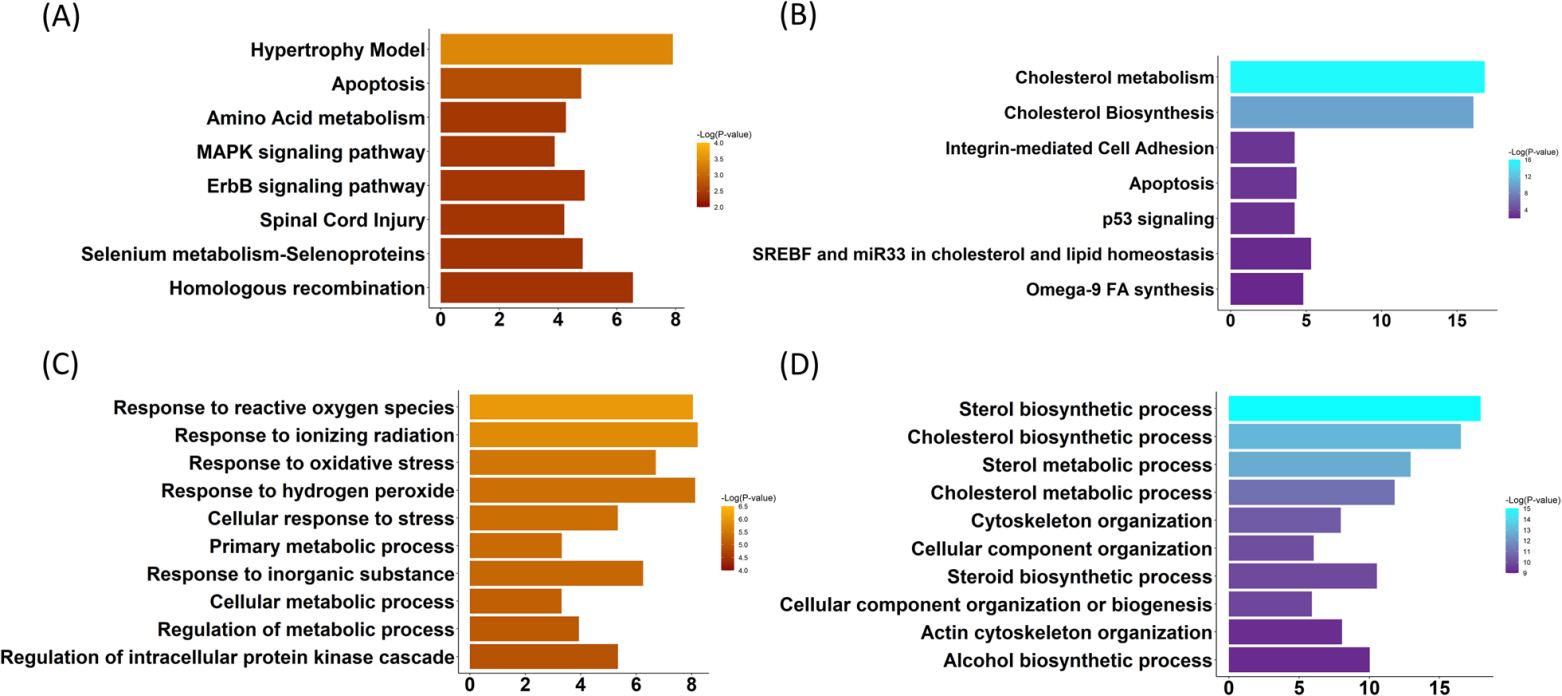
Signatures of the common cardiomyocyte response to *Trypanosoma cruzi* infection. **A**, **B** ‘WikiPathways’ and **C**, **D** ‘Biological Process gene ontology Terms’ enriched by commonly **A**, **C** upregulated and **B**, **D** downregulated genes of the host cardiomyocyte at 24-h post-infection are sorted based on *P*-value. Bar lengths represent enrichment Z-scores

The 107 upregulated DEGs shared among all three infection conditions enriched pathways related to ‘Hypertrophy model’ (*Z* = 7.90, *P* < 0.001), ‘Apoptosis’ (*Z* = 4.8 *P* < 0.01), ‘Aminoacid metabolism’ (*Z* = 4.3, *P* < 0.01), ‘MAPK signaling pathway’ (*Z* = 3.9, *P* < 0.01), ‘ErbB signaling pathway’(*Z* = 4.9, *P* < 0.01), ‘Spinal cord injury’ (*Z* = 4.2, *P* < 0.01), ‘Selenium metabolism-selenoproteins’ (*Z* = 4.8, *P* < 0.01), and ‘Homologous recombination’ (*Z* = 6.5, *P* < 0.01) (Fig. 7A). The same gene list was enriched in genes associated with the Biological Process GO terms of ‘Response to reactive oxygen species’ (*Z* = 8.1, *P* < 0.001), ‘Response to ionizing radiation’ (*Z* = 8.2, *P* < 0.001), ‘Response to oxidative stress’ (*Z* = 6.7, *P* < 0.001), ‘Response to hydrogen peroxide’ (*Z* = 8.1, *P* < 0.001), ‘Cellular response to stress’ (*Z* = 5.3, *P* < 0.001), ‘Primary metabolic process’ (*Z* = 3.3, *P* < 0.001), ‘Regulation of metabolic process’ (*Z* = 3.9, *P* < 0.001), and ‘Regulation of intracellular protein kinase cascade’ (*Z* = 5.4, *P* < 0.001) (Fig. 7C).

Of the 315 commonly downregulated genes, 14 had an FC ≤ 0.2 (Fig. 6B). The 315 downregulated DEGs shared among all three infection conditions enriched pathways related to ‘Cholesterol metabolism’ (*Z* = 16.8, *P* < 0.001), ‘Cholesterol biosynthesis’ (*Z* = 16.1, *P* < 0.001), ‘Integrin-mediated cell adhesion’ (*Z* = 4.2, *P* < 0.01), ‘Apoptosis’ (*Z* = 4.3, *P* < 0.01), ‘p53 signaling’ (*Z* = 4.2, *P* < 0.01), ‘SREBF and miR33 in cholesterol and lipid homeostasis’ (*Z* = 5.3, *P* < 0.01) and ‘Omega-9 FA synthesis’ (*Z* = 4.8, *P* < 0.01) (Fig. 7B). The same gene list was enriched in genes associated with the Biological Process GO terms of ‘Sterol biosynthetic process’ (*Z* = 17.9, *P* < 0.001), ‘Cholesterol biosynthetic process’ (*Z* = 16.5, *P* < 0.001), ‘Sterol metabolic process’ (*Z* = 12.9, *P* < 0.001), ‘Cholesterol metabolic process’ (*Z* = 11.8, *P* < 0.001), ‘Cytoskeleton organization’ (*Z* = 7.9, *P* < 0.001), ‘Cellular component organization’ (*Z* = 6.0, *P* < 0.001), ‘Steroid biosynthetic process’ (*Z* = 10.5, *P* < 0.001), ‘Cellular component organization or biogenesis’ (*Z* = 5.9, *P* < 0.001), ‘Actin cytoskeleton organization’ (*Z* = 8.1, *P* < 0.001), and ‘Alcohol biosynthetic process’ (*Z* = 10.0, *P* < 0.001) (Fig. 7D).

**Fig. 8:**
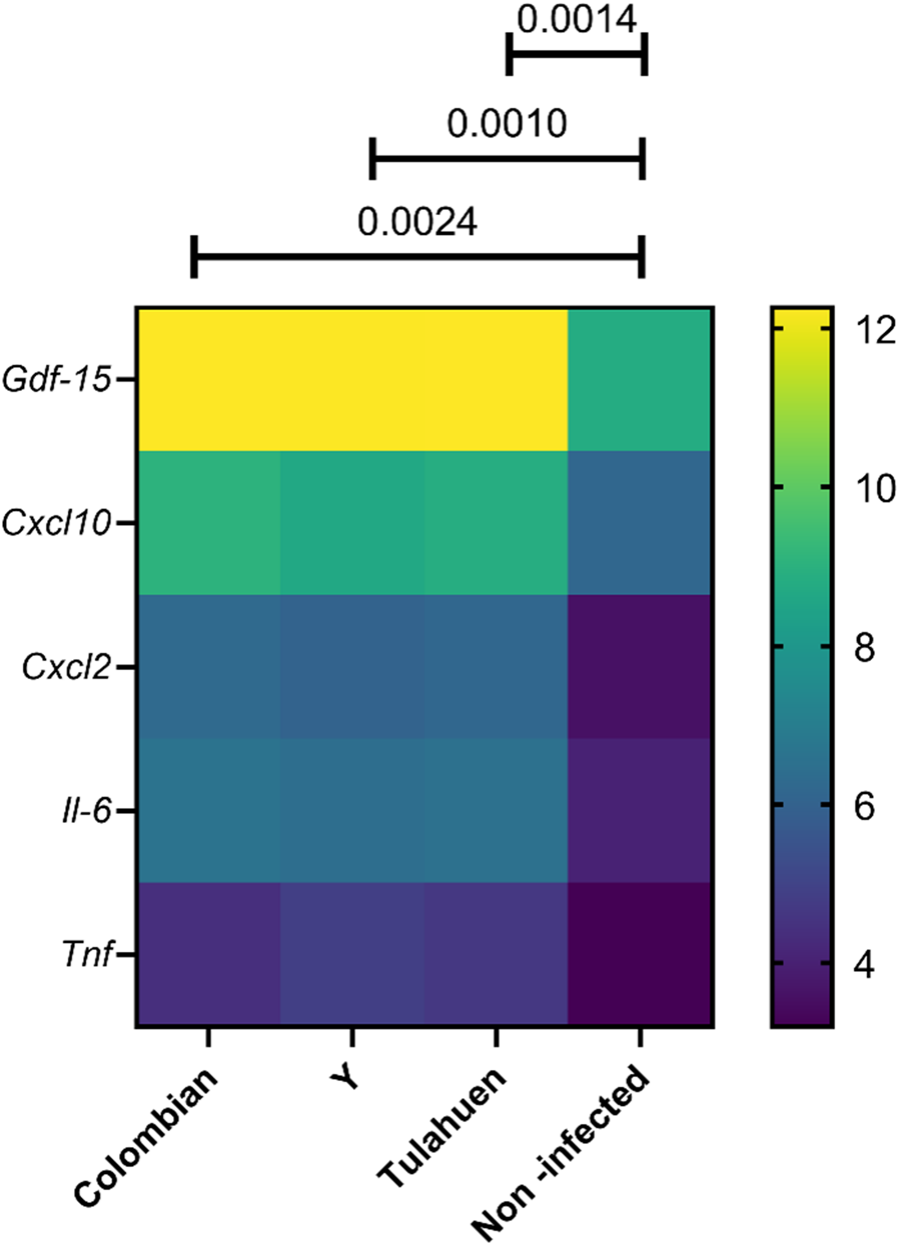
Heatmap of expression levels of inflammation-related genes in HL-1 cells infected *with T. cruzi* at 24-h post-infection. Genes are represented in rows and infection conditions are represented in columns. Color legend represents the logarithm 2 of gene expression level. *P*-values were calculated by paired T-test. *Gdf-15* growth differentiation factor 15, *Cxcl10* C-X-C motif chemokine ligand 10, *Cxcl2* C-X-C motif chemokine ligand 2, *Il-6* interleukin-6, *Tnf* tumor necrosis factor-alpha

A biased search of inflammation-related genes returned five cytokines and chemokines that were differentially expressed among all three cardiomyocyte infection conditions, namely *Gdf-15*, *Cxcl10*, *Cxcl2*, *Il-6* and *Tnf.* The expression levels of these inflammatory cytokines were significantly higher in the three cardiomyocyte infection conditions compared to the non-infected control: Colombian strain *P* = 0.0024, Y strain *P* = 0.001 and Tulahuen strain* P* = 0.0014 (Fig. 8).

### Transcriptomic remodeling unique to Colombian- and Y-infections

**Fig. 9:**
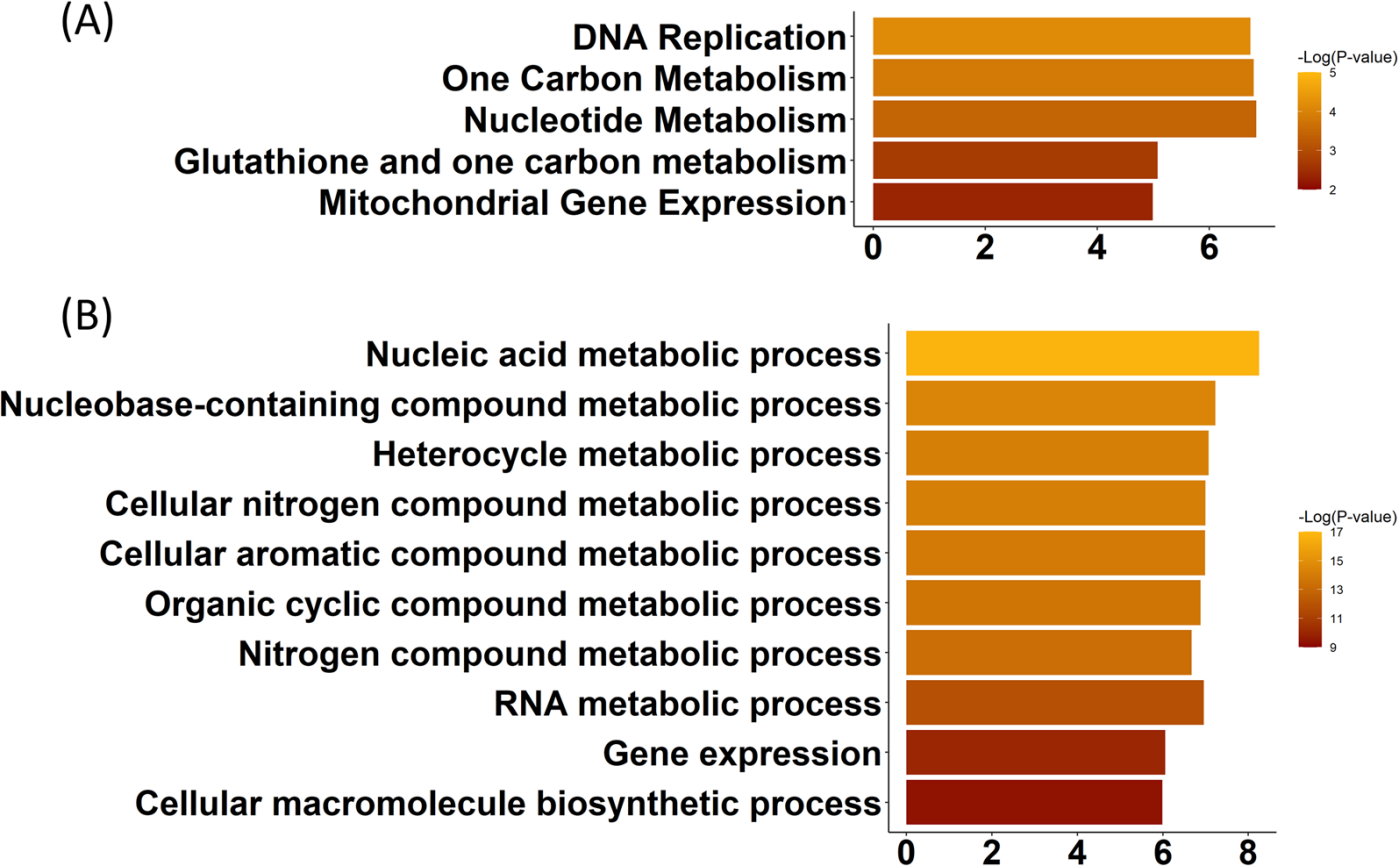
Signatures of transcriptomic remodeling unique to Colombian- and Y-infections. ‘WikiPathways’ (**A)** and 'Biological Process gene ontology Terms’ (**B)**, enriched by the genes uniquely upregulated at 24-h post-infection in Colombian- and Y-infected cardiomyocytes are sorted based on *P*-value. Bar lengths represent enrichment Z-scores

Upregulated genes over-represented the DEGs shared among the Colombian- and Y-infected conditions and thus formed the focus of our interest. Such 331 genes enriched pathways related to ‘DNA replication (*Z* = 6.8, *P* < 0.001)’, ‘One Carbon metabolism (*Z* = 6.8, *P* < 0.001)’, ‘Nucleotide metabolism (*Z* = 6.8, *P* < 0.001)’, ‘Glutathione and one-carbon metabolism (*Z* = 5.1, *P* < 0.01)’, and ‘Mitochondrial gene expression (*Z* = 4.9, *P* < 0.01)’ (Fig. 9A). The same gene list was enriched in genes associated with the Biological Process GO terms of ‘Nucleic acid metabolic process’ (*Z* = 8.3, *P* < 0.001), ‘Nucleobase-containing compound metabolic process (*Z* = 7.2, *P* < 0.001), ‘Heterocycle metabolic process’ (*Z* = 7.1, *P* < 0.001), ‘Cellular nitrogen compound metabolic process’ (*Z* = 6.9, *P* < 0.001), ‘Cellular aromatic compound metabolic process’ (*Z* = 6.9, *P* < 0.001), ‘Organic cyclic compound metabolic process’ (*Z* = 6.8, *P* < 0.001), ‘Nitrogen compound metabolic process’ (*Z* = 6.7, *P* < 0.001), ‘RNA metabolic process’ (*Z* = 6.9, *P* < 0.001), and ‘Gene expression’ (*Z* = 6.1, *P* < 0.001) (Fig. 9B).

Differentially expressed genes corresponding to the ‘Glutathione and one-carbon metabolism’ pathway (Wikipathway ID: WP730) were individually tracked and analyzed, where the genes corresponding to the ‘one-carbon metabolism’ and ‘Glutathione metabolism’ showed an up and downregulation trend, respectively. Shared, as well as Colombian/Y-unique, pathways and GO terms enriched with upregulated DEGs upon infection are summarized in Table 1.

**Table 1:** Summarization of the pathways enriched by the upregulated genes in all the *T. cruzi-infection* conditions

	Pathway name	Common	Exclusive to Colombian/Y strain
Upregulated WikiPathways	DNA replication		O
	One carbon metabolism		O
	Glutathione and one-carbon metabolism		O
	Nucleotide metabolism		O
	Mitochondrial gene expression		O
	Hypertrophy model	O	
	Homologous recombination	O	
	Apoptosis	O	
	ErbB signaling pathway	O	
	Selenium metabolism selenoproteins	O	
	Amino acid metabolism	O	
	Spinal cord injury	O	
	MAPK signaling pathway	O	
Upregulated biological process GO terms	Nucleic acid metabolic process		O
	Nucleobase-containing compound metabolic process		O
	Heterocycle metabolic process		O
	Cellular nitrogen compound metabolic process		O
	Cellular aromatic compound metabolic process		O
	Organic cyclic compound metabolic process		O
	Nitrogen compound metabolic process		O
	RNA metabolic process		O
	Gene expression		O
	Response to reactive oxygen species	O	
	Response to ionizing radiation	O	
	Response to oxidative stress	O	
	Response to hydrogen peroxide	O	
	Cellular response to stress	O	
	Primary metabolic process	O	
	Response to inorganic substance	O	
	Regulation of metabolic process	O	
	Regulation of intracellular protein kinase cascade	O	

### Transcriptomic remodeling unique to Tulahuen-infections

Tulahuen-infection led to the unique downregulation of 218 DEGs in the cardiomyocyte transcriptome. Significantly enriched pathways were: ‘Proteosome degradation’ (*Z* = 5.1, *P* < 0.001), ‘Electron transport chain’ (*Z* = 4.1, *P* < 0.01), and ‘Oxidative phosphorylation’ (*Z* = 3.7, *P* < 0.01). Top 10 Biological processes GO terms enriched by this gene set are ‘Cellular metabolic process’ (*Z* = 3.9, *P* < 0.001), ‘Metabolic process’ (*Z* = 3.4, *P* < 0.001), ‘Cellular process’ (*Z* = 2.3, *P* < 0.001), ‘Organic substance metabolic process’ (*Z* = 3.2, *P* < 0.001), ‘Localization’ (*Z* = 4.0, *P* < 0.001), ‘Organic substance transport’ (*Z* = 4.5, *P* < 0.001), ‘Macromolecule localization’ (*Z* = 4.4, *P* < 0.001), ‘Cellular protein metabolic process’ (*Z* = 3.9, *P* < 0.001), and ‘Cellular protein metabolic process’ (*Z* = 3.9, *P* < 0.001).

### *Trypanosoma cruzi* infection increases intracellular reactive oxygen species in HL-1 cardiomyocytes

**Fig. 10:**
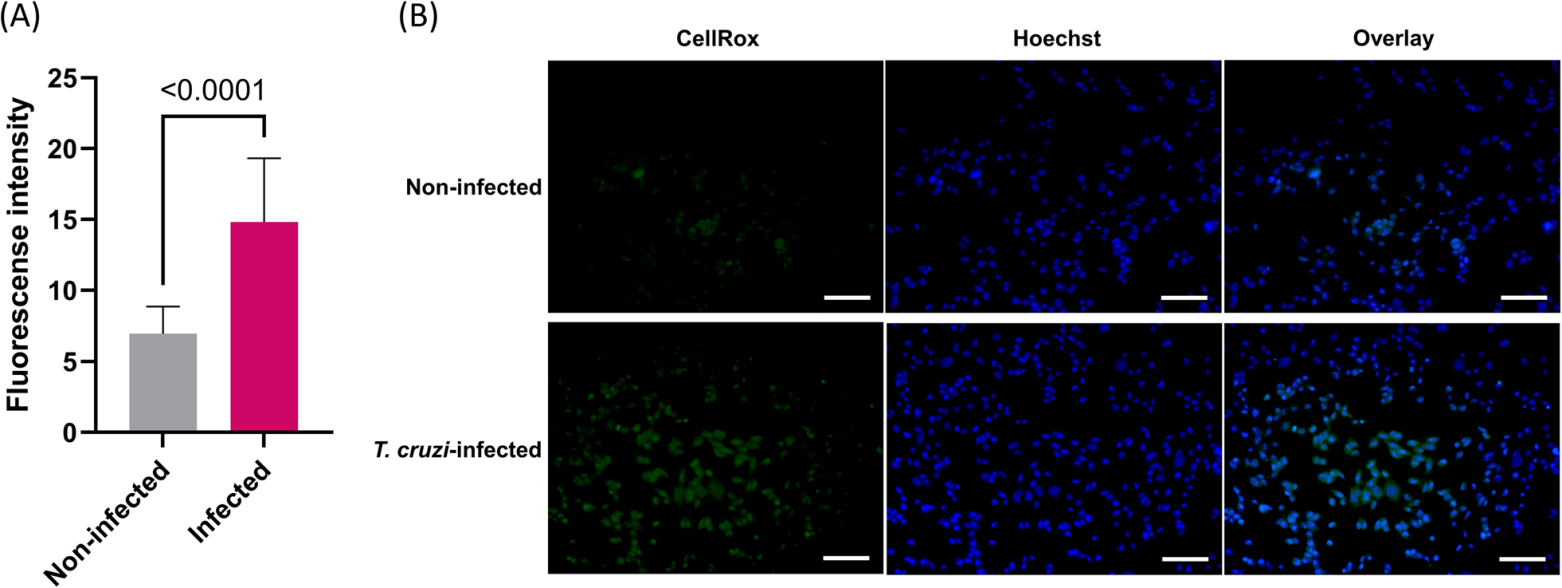
Elevated intracellular levels of reactive oxygen species in *Trypanosoma cruzi*-infected cardiomyocytes. **A** Signal intensity of reactive oxygen species-specific fluorescence was higher in the cardiomyocytes infected with the Y strain at 24-h post-infection, compared with non-infected control cells. Statistical comparison was performed with Mann–Whitney test. Bars represent mean fluorescence intensity and error bars represent standard deviation. **B** Representative fluorescence microscopy images from reactive oxygen species-specific CellRox green staining. Comparison with the non-infected (upper) control cells shows higher intensity of reactive oxygen species fluorescence signal in the cardiomyocytes 24-h post-infection with *Trypanosoma cruzi* Y strain (lower). Scale bars represent 100 µm

With specific interest in the accentuated ROS mitigation response elucidated from transcriptomic analysis of the cellular models, we next evaluated the intracellular ROS level of infected cardiomyocytes. HL-1 cardiomyocytes showed higher ROS levels upon infection with *T. cruzi* in comparison to non-infected controls. Signal intensity (mean, interquartile range) of the ROS-specific fluorescent dye, CellRox green, was significantly higher in the *T. cruzi*-infected condition (16.55, 9.73–19.02; 6.85, 5.70–7.54; *P* < 0.0001), further validating the relevant role of ROS in CCM pathogenesis (Fig. 10A). Representative images are shown in Fig. 10B.

## Discussion

In vitro infection using HL-1 cardiomyocytes and *T. cruzi* from different genetic strains provided mechanistic clues to CCM progression.

Subsequent transcriptomic and functional analyses of the in vitro cardiomyocyte infection models had characterized in-depth the myocardial innate response that is a potential determinant of CCM progression. The cross-strain comparative analysis led to the characterization of the universal hallmarks of the cardiomyocyte response against *T. cruzi* infection, consisting of DEGs evoked under all three infection conditions. Among the hallmark transcriptomic signatures were pathways related to oxidative stress (the ‘Response to reactive oxygen species’, ‘Response to oxidative stress’, ‘Response to hydrogen peroxide’ GO terms), cell proliferation and cardiac hypertrophy (the ‘ErbB signaling’, ‘Hypertrophy model’ and ‘MAPK signaling’ pathways), and those related to heart energy metabolism (the ‘SREBF and miR33 in cholesterol and lipid homeostasis’, and ‘Omega-9 FA synthesis’ pathways and the ‘Sterol biosynthetic process’, ‘Cholesterol biosynthetic process’, ‘Sterol metabolic process’, ‘Cholesterol metabolic process’ and ‘Steroid biosynthetic process’ GO terms).

Firstly, possible derangements in cardiomyocyte ROS homeostasis, as observed here in our cellular model, has been previously reported to accompany cardiac infection with T. cruzi [27, 29, 31, 38–40]. ROS mitigation is not a unique host response but in fact a shared mechanism of the cardiomyocyte against the more common insults leading to dilated cardiomyopathy, namely ischemia/hypoxia and Coxsackie virus infection [41–43]. Among the unique remodeling patterns in Colombian and Y infections were the upregulation of glutathione metabolism for ROS mitigation in CCM progression. Previous in vitro [23, 24] and in vivo studies [22] have shown that glutathione and its related metabolites serve as key antioxidants in cardiomyocytes under infectious or hypoxic stress [44–46]. Altogether, the concomitant upregulation of the compensatory ‘Glutathione and one-carbon metabolism’ pathways in Colombian- and Y-infected cardiomyocytes further highlights the intriguing role of ROS homeostasis in CCM pathogenesis. Several genes from the ‘Hypoxia signaling’ pathway, notably the hypoxia-inducible factor 1-α (HIF-1α) have been reported to be upregulated in human cardiomyocytes as a response to infection with T. cruzi [18]. HIF-1α has been reported to be positively correlated with myocarditis severity in patients with CCM [47]. Although in our dataset the ‘hypoxia signaling’ pathway was not enriched, a detailed search of HIF-1α-targeted genes revealed the downregulation of *Glutathione peroxidase 3* (*Gpx3)*, and the upregulation of *Growth Arrest and DNA Damage Inducible Alpha (Gadd45a)*. An overtime decline of glutathione peroxidase levels has been previously described in a CCM model after an initial burst on its levels triggered by chronic ROS accumulation typical of hypoxic states [48], and might promote progression to heart failure [49].

Secondly, upregulation of cell proliferation pathways, such as ‘PI3K/AKT’, ‘Extracellular signal-regulated kinases 1 and 2 (ERK1/2)’, ‘JAK/STAT signaling’ and ‘The mammalian target of Rifampicin (mTORC)’ pathways, have been shown previously in multiple studies with human and murine cell lines infected with a variety of *T. cruzi* strains [24, 29, 50–59]. Cell proliferation seems a shared response mechanism among variable host cells, considering the differential expression of 18 well-known pro-proliferative genes, including *Rhob*, *Atf3*, *Brca2*, *Jun*, *Jund*, *Myc*, as well as the enrichment in the mutually interacting pathways of ErbB and MAPK signaling, observed in the cardiomyocyte response of our data set [56]. A summarization of the DEGs of the ErBb and MAPK signaling pathways is depicted in Additional file 2: Fig. S3. In the context of cardiomyocytic responses, although initially adaptive to stress stimuli, such signaling pathways are the underlying cause of pathological cardiac hypertrophy and mechanical dysfunction when chronically activated [60–62]. Thus, these pathways have constituted the ‘double-edged' roles in the development of, and the common potential targets for, ischemic and viral cardiomyopathies [63–65].

Thirdly, cellular metabolism can drastically influence cellular functions in the failing heart [66, 67]. Previous in vitro studies have replicated downregulation of key genes in lipid metabolism such as *Dhcr24* [23, 68]. The uncoupling of heart lipid synthesis and β-oxidation, coined cardiac lipidopathy, has been suggested as a hallmark metabolic feature of CCM [69]. Sterol regulatory element binding factor (*Srebf*), a transcription factor that regulates cellular lipid homeostasis by controlling the expression of an array of enzymes required for endogenous cholesterol, fatty acid, triacylglycerol, and phospholipid synthesis [70, 71] was found to be one of the commonly downregulated DEGs in our dataset at 24 and 48 hpi (Additional file 1). *Srebf* has been proposed as a potential target for ameliorating the cardiac lipidopathy in Chagas disease [72, 73]. This imbalance of lipid synthesis and beta-oxidation became further prominent in our in vitro model at 48 hpi, accompanied by the downregulation of ‘Fatty acid oxidation’ and ‘Beta-oxidation’ pathway-related genes: *Acss2*, *Acsl4*, and *Acsl5* (Additional file 1). The correction of such pathogenic metabolic uncoupling that may potentiate *T. cruzi* proliferation may exert therapeutic potential against CCM through the restoration of metabolic homeostasis [73–75]. While the glycolysis pathway was not significantly enriched in our dataset, a manual search of glucose metabolism-related genes showed the common upregulation of *Solute Carrier Family 2 Member 3*. The upregulation of *6-phosphofructo-2-kinase/fructose-2,6-biphosphatase 3* and *Phosphoglycerate mutase family member 5* in Colombian and Y-infected cells only*;* and the downregulation of *Solute Carrier Family 2 Member 1* and P*hosphofructokinase, liver, B-type* in Tulahuen infection. Noteworthy, *Arrestin Domain Containing 4* (*Arrdc4*), *Thioredoxin interacting protein* (*Txnip),* and *Activating transcription factor 3* (*Atf3)*, which were among the most extensively upregulated genes common to all cardiomyocyte infection conditions, may hinder glucose uptake and lead to apoptosis in vitro as shown in murine cardiomyocytes and *T. cruzi*-infected human fibroblasts [76–80]. Their upregulation in vivo promotes cardiac hypertrophy and contractile dysfunction [81, 82]. Furthermore, *Atf3*, which is induced by ROS and pathogens including *T. cruzi, m*ay promote fibrosis and cardiac hypertrophy [83–89]. *T. cruzi*-infected Induced Pluripotent Stem Cardiomyocytes (iPS-CM) display an activation of the glycolysis and oxidative phosphorylation pathway where attenuation of glucose uptake significantly decreased parasites’ invasion and replication within the host [18]. The aforementioned phenomenon has also been observed after restricting the cell from other carbon sources like glutamine [90]. This number of phenotypic effects derived from their dysregulation renders them an interesting group of genes to explore their role in the pathogenesis of CCM. Further functional analysis in vitro on actual glucose utilization using our cellular model may elucidate whether a metabolic compensatory mechanism is operating to mitigate the ongoing infection.

Additionally, among the top upregulated DEGs was the TGF-β superfamily *Gdf15*, an emerging cardiovascular biomarker. GDF15 protects cardiomyocytes from ROS- and nitric oxide-induced apoptosis in vitro. In addition, the risk stratification potential of GDF15 in patients with heart failure is currently gaining attention [91–93]. Given that the upregulation of GDF15 comprises the cardinal feature of cardiomyocyte response in our in vitro model, the behavior of this cytokine in actual CCM patients arises as our next intriguing research question.

Regional factors, including circulating *T. cruzi* strains, have attracted attention as likely determinants of Chagas disease phenotypic expression and CCM progression [94–99]. Experimental data from murine and cellular models of CCM have shown that *T. cruzi* Colombian and Y strains among others cause severe myocarditis and higher mortality [22, 100, 101]. Epidemiological data from Colombian, Brazilian and Argentinian patients suggest that CCM prevalence and severity is increased in those infected with certain *T. cruzi* strains [102–105]. Although not the only causative factor, it is likely that infections caused by parasites of specific genetic traits render the host prone to severe CCM forms.

The limitations of this study include the lack of normalization of the gene expression values by intracellular amastigote burden across the infecting *T. cruzi* strains. Higher infectivity was observed in the Colombian and Y strains compared with the Tulahuen strain and may have affected the time course of progression of the in vitro pathogenesis. Such a trend towards less infectivity of the Tulahuen strain has been observed in previous studies [106, 107]. Single-cell resolution analyses, to overcome the weaknesses of bulk transcriptomics, may aid in eliminating potential interpretation biases of the observed inter-strain differences from the current approach. It is also noteworthy that since the present study was mainly observational in nature, further functional experiments are to be performed in order to make conclusions about the relevance of the individual pathways that were highlighted here.

## Conclusions

In conclusion, the ubiquitously observed hypertrophic and oxidative stress-related transcriptome remodeling are universal hallmarks of the cardiomyocyte response against *T. cruzi* infection. The Colombian and Y strain elicited an accentuated response in cardiomyocyte ROS mitigation, nitrogen, and glutathione metabolism suggesting a role in CCM progression. The downregulation of the cardiomyocyte’s energy metabolic pathways exclusively observed in the Tulahuen strain infection may enact a protective effect against the infection.

### Supplementary Information

**Additional file 1: **Valid gene set at 24 and 48 hpi. Valid gene probes above the thresholds of intensity in every infection condition at 24- and 48-hpi timepoints.

**Additional file 2: Figure S1**. Representative images of *Trypanosoma cruzi-*infected cardiomyocytes reflecting infectivity rate differences among strains. Diff-Quick staining. Scale bars represent 50 µm. **Figure S2.** Remodeling of gene expression profiles in cardiomyocytes treated with Tumor Necrosis Factor-α (Positive control) at 24- and 48-h post-infection (hpi). Y axes indicate the gene expression level of the non-infected control cardiomyocyte, while X-axes indicate gene expression levels of Tumor Necrosis Factor-treated cardiomyocytes. The black dashed line, and upper and lower red dashed lines show the line of identity, and the 0.5- and 2-fold-change thresholds, respectively. Each dot represents a differentially expressed gene (DEG). Blue and pink shaded areas represent down- and up-regulated DEGs, respectively. **Figure S3.** Cytoscape network visualization of the *Mus musculus* ‘ErBb-’ and ‘MAPK signaling’ pathways. Network showing the commonly upregulated genes of both pathways (orange nodes) and their two closest neighbors (light blue nodes). Red and purple circles enclose genes that belong to the ‘ErBb-’ and ‘MAPK signaling’ pathways, respectively. Edges (black lines) represent links between genes.

## Data Availability

The data that support the findings of this study are openly available in Mendeley Data, at http://doi.org/10.17632/v82vdkt7ws.2

## Abbreviations

*Arrdc4*: *Arresting domain containing 4*
*Atf3*: *Activating transcription factor 3*
CCdT: Cultured cell-derived trypomastigotes
CCM: Chagas cardiomyopathy
DAS: Data availability statement
DEG: Differentially expressed genes
FC: Fold change
*Gdf15*: Growth/differentiation factor 15
GO: Gene ontology
hpi: Hours post-infection
LIT: Liver infusion tryptose
MOI: Multiplicity of infection
*P*: *P*-Value
PBS: Phosphate buffered saline
PCA: Principal component analysis
ROS: Reactive oxygen species
*T. cruzi*: *Trypanosoma cruzi*
*Tnf*: *Tumor necrosis factor*
TNF-α: Tumor necrosis factor α
*Txnip*: Thioredoxin interacting protein
Z: Z-score
