# Supplementary figures and images for: Differential cardiomyocyte transcriptomic remodeling during in vitro Trypanosoma cruzi infection using laboratory strains provides implications on pathogenic host responses
Source: Trop Med Health. 2023 Dec 7;51:68. doi: 10.1186/s41182-023-00552-6 (PMC10702087; doi:10.1186/s41182-023-00552-6)

**Additional file figures**

**Figure S1.**

**
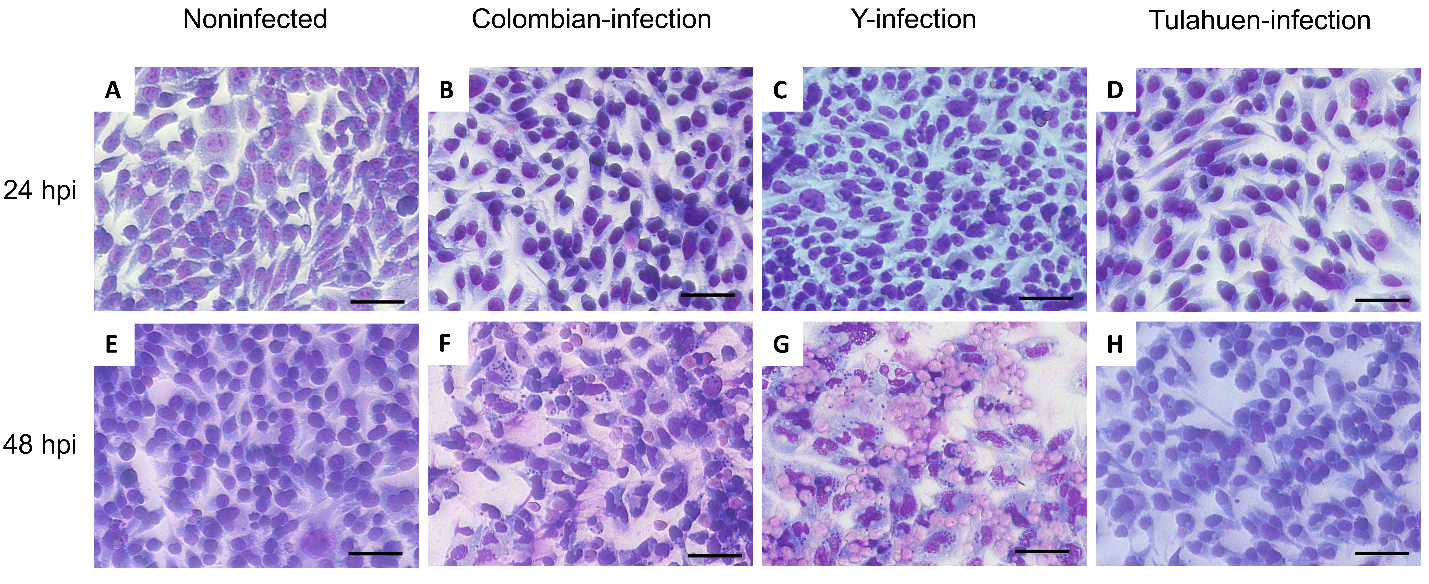
**

**Figure S2**

**
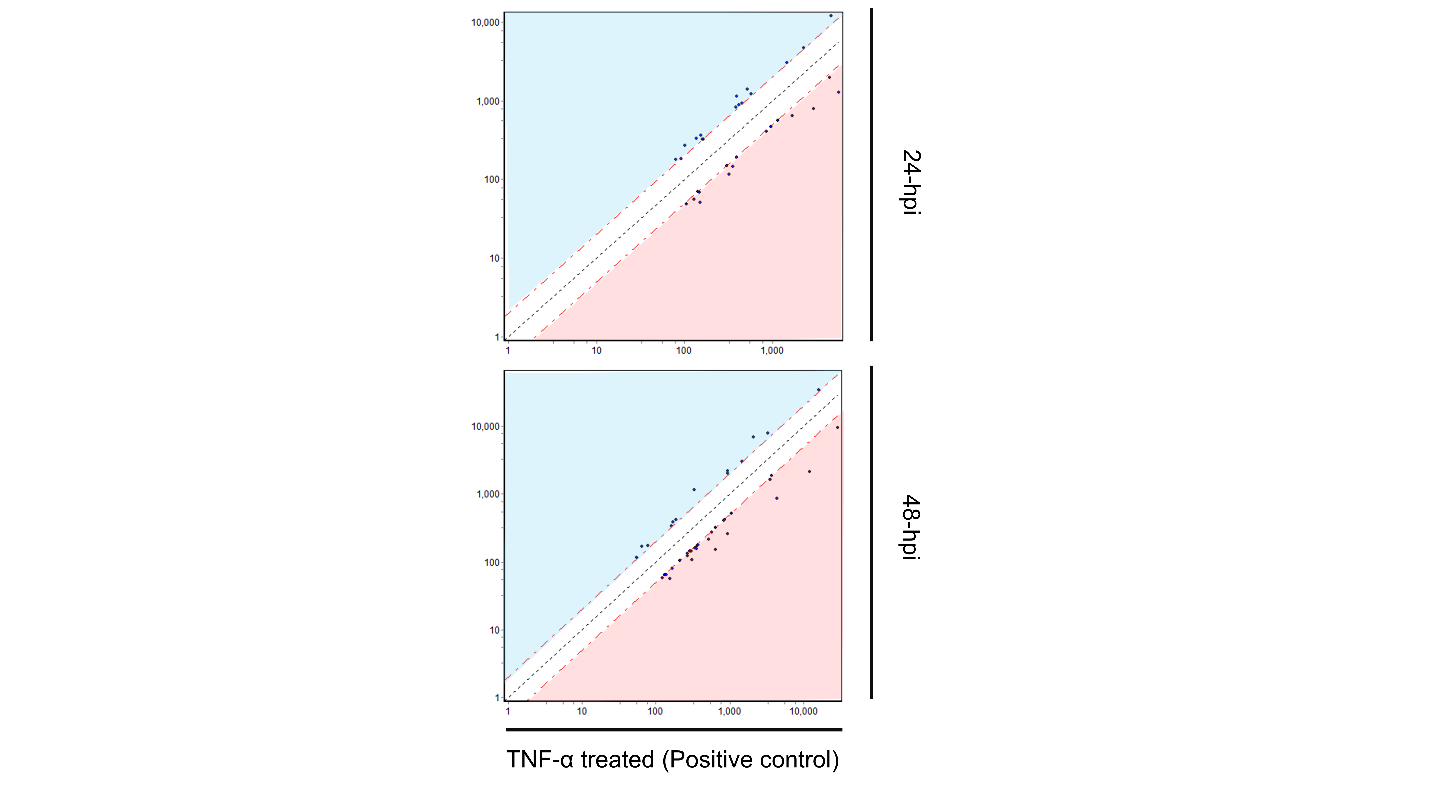
**

**Figure S3**

**
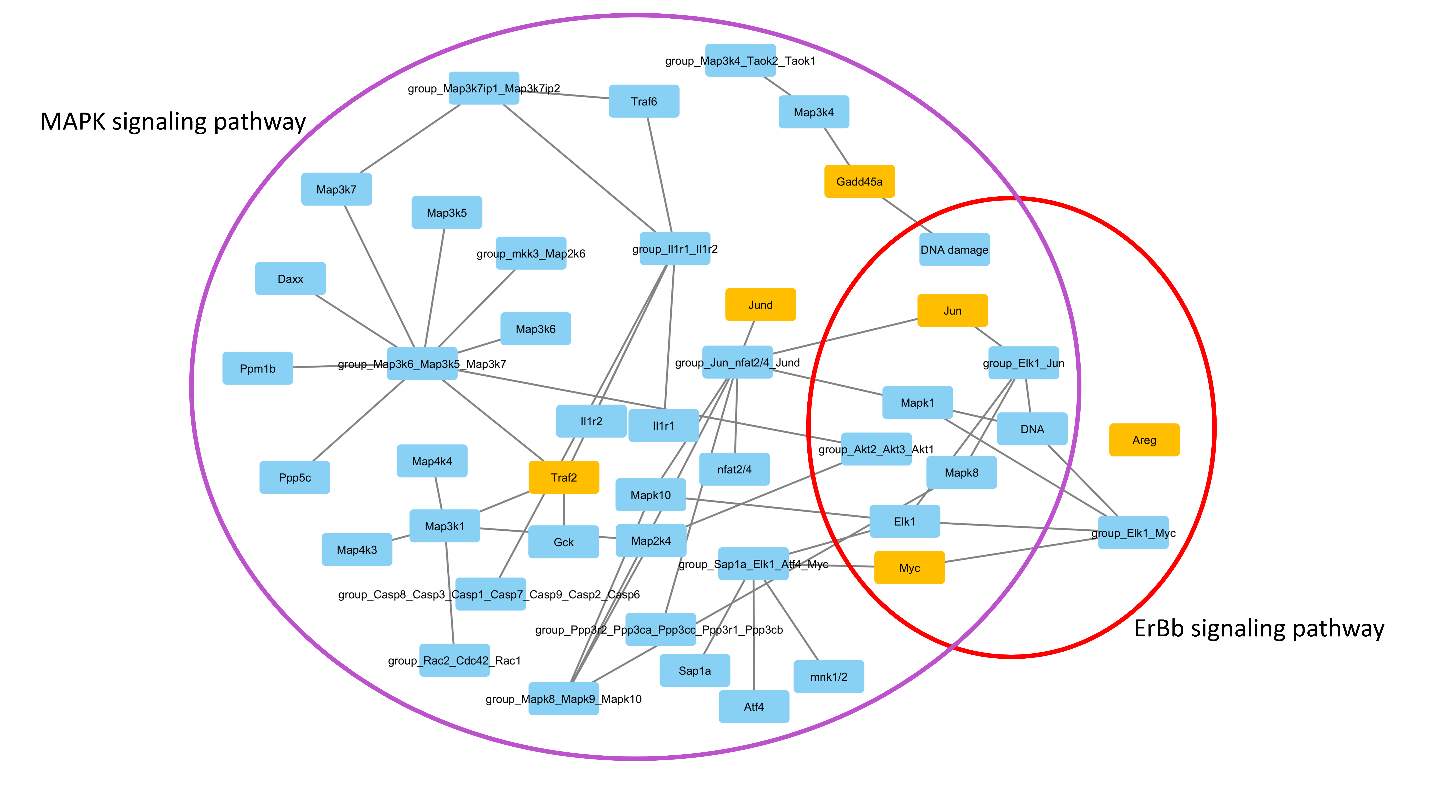
**

Supplement: Supplementary file 2 — Additional file 2: Figure S1. Representative images of Trypanosoma cruzi-infected cardiomyocytes reflecting infectivity rate differences among strains. Diff-Quick staining. Scale bars represent 50 µm. Figure S2. Remodeling of gene expression profiles in cardiomyocytes treated with Tumor Necrosis Factor-α (Positive control) at 24- and 48-h post-infection (hpi). Y axes indicate the gene expression level of the non-infected control cardiomyocyte, while X-axes indicate gene expression levels of Tumor Necrosis Factor-treated cardiomyocytes. The black dashed line, and upper and lower red dashed lines show the line of identity, and the 0.5- and 2-fold-change thresholds, respectively. Each dot represents a differentially expressed gene (DEG). Blue and pink shaded areas represent down- and up-regulated DEGs, respectively. Figure S3. Cytoscape network visualization of the Mus musculus ‘ErBb-’ and ‘MAPK signaling’ pathways. Network showing the commonly upregulated genes of both pathways (orange nodes) and their two closest neighbors (light blue nodes). Red and purple circles enclose genes that belong to the ‘ErBb-’ and ‘MAPK signaling’ pathways, respectively. Edges (black lines) represent links between genes. [file 41182_2023_552_MOESM2_ESM.docx]
